# Value of computed tomography texture analysis for prediction of perioperative complications during laparoscopic partial nephrectomy in patients with renal cell carcinoma

**DOI:** 10.1371/journal.pone.0195270

**Published:** 2018-04-18

**Authors:** Georg Bier, Simone Bier, Malte Niklas Bongers, Ahmed Othman, Ulrike Ernemann, Johann-Martin Hempel

**Affiliations:** 1 Department of Neuroradiology, Eberhard-Karls-University Tuebingen, Tuebingen, Germany; 2 Department of Urology, Eberhard-Karls-University Tuebingen, Tuebingen, Germany; 3 Department of Diagnostic and Interventional Radiology, Eberhard-Karls-University Tuebingen, Tuebingen, Germany; University of Campinas, BRAZIL

## Abstract

**Purpose:**

Tumorous texture is a marker for tumor tissue inhomogeneity. Based on this assumption, this study aims to evaluate the value of computed tomography texture analysis for imaging-based prediction of perioperative complications during laparoscopic partial tumor nephrectomy.

**Methods:**

A total of 106 patients with histologically confirmed renal cell carcinoma and pre-operative CT were included and volumetric texture analysis of the tumors was performed by two readers. Texture analysis parameter ratios and differences were calculated using the kidney parenchyma as reference (“reference-corrected”). Regression analysis was performed, regarding the value of the texture analysis parameters, for assessment of the tumor nuclear grade and the prediction of peri- and postoperative complications and approximated blood loss. Moreover, the inter-rater agreement in terms of the intra-class correlation coefficient (ICC) was calculated.

**Results:**

Regarding the reference-corrected values, the predictive value of texture analysis parameters for severe perioperative complications was highest for the standard deviation of the mean attenuation (Area under curve/AUC, .615; sensitivity, 93.8%, specificity, 30.0%), followed by the uniformity (AUC, .599; sensitivity, 62.5%, specificity, 60.0%), and the uniformity of distribution of positive pixels (AUC, .597; sensitivity, 62.5%; specificity, 61.1%).

Regarding the blood loss, the uniformity of positive pixel values (UPP; AUC, 0.638), uniformity (AUC, 0.635), and entropy (AUC, 0.633) yielded the best predictive values, whilst the tumor grade was a weaker predictor (AUC, 0.574).

The applied texture analysis parameters did not correlate with the time of surgery or the warm ischemic time. All measured parameters were better predictors for complications than the tumor diameter alone. The inter-rater agreement was almost perfect (ICC, .982).

**Conclusion:**

CT and CT texture analysis parameters are valuable for prediction of perioperative outcome before laparoscopic partial nephrectomy in patients with renal cell carcinoma.

## Introduction

Surgery of renal cell carcinoma (RCC) via laparoscopic partial nephrectomy (LPN) is a common technique, especially for small tumors [1]. However, the laparoscopic approach can be accompanied by potentially severe complications, in particular hemorrhage, formation of pseudo aneurysms or urine leakage [2, 3]. Surgical outcome is significantly influenced by the tumor localization and environment, which has led to several predictive imaging-based scoring systems [4]. Especially the R.E.N.A.L. nephrometry scoring system has been identified as a valuable, predictive tool for pretherapeutic assessment of surgical risk [4, 5]. R.E.N.A.L. is mainly based on tumor size and localization, whilst the tumorous internal structure is not taken into consideration. Although, the tumor microstructure, especially tumorous angiogenesis, can be a complicating factor when it comes to intraoperative blood loss [6, 7] and therefore is important.

It was recently demonstrated, that the structural inhomogeneity of RCCs could be assessed noninvasively by the application of modern computed tomography (CT) texture analysis techniques. This technique has already been proposed for estimation of the tumor dignity, the tumor grade, and for therapy response assessment [8–12]. However, the complexity of surgery is increasing with the macroscopic tumor structure itself. Therefore, texture analysis may also reveal structural differences of a tumor meaning a different tumor “haptic” or stickiness during surgery. Moreover, inhomogenous hyperdense tumor structures may reflect hypervascularized tumor areas with increased microvascular density (MVD), which is known as a stronger predictor than the nuclear grade and may also imply an increased perioperative risk, especially regarding tumor bleedings [13]. Regarding this, the question arises whether CT texture analysis could also be useful to assess the internal tumor structure and predict perioperative complications.

Therefore, the purpose of this study was to retrospectively evaluate morphological CT parameters including texture analysis and their predictive value regarding peri- and postoperative complications, time of surgery, blood loos and warm ischemic time in patients, which underwent LPN due to renal cell carcinoma.

## Materials and methods

### Study design and patient selection

**Fig 1:**
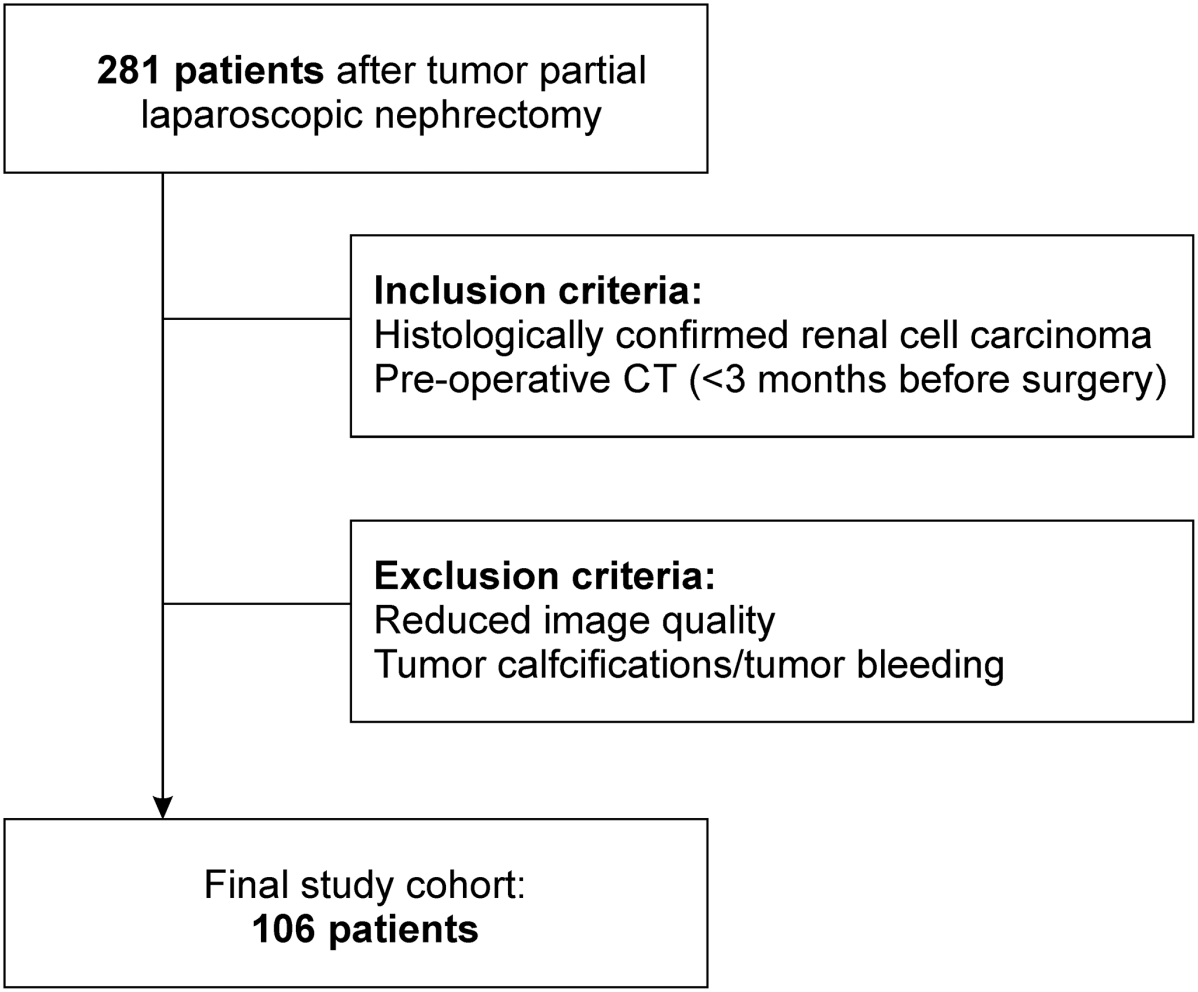
Flow chart of the patient selection.

This study was designed as a retrospective study, it was reviewed and approved by our local ethics committee with a waiver for informed consent (Ethics committee of the medical faculty of the Eberhard Karls University Tübingen, Germany; internal reference number: 078/2016BO2). A total number of 281 consecutive patients that received laparoscopic partial nephrectomy between 02/2005 and 11/2013 at our department of Urology, were selected from a recently examined study cohort (see Bier et al. [4]). A flow chart representing the patient selection process is given in Fig 1. Primary selection criterion was the histological confirmation of a renal cell carcinoma after surgery. Moreover, only patients that received a computed tomography <3 months afore surgery with venous or portovenous contrast phase, were included. Exclusion criteria were insufficient image quality and macroscopic tumor calcifications as well as acute tumor bleeding, identified on these CT images. Patients were sub-classified regarding the histological tumor type (clear cell RCC vs. non-clear cell RCC) and the nuclear tumor grade.

The approximated blood loss during surgery was assessed based on the underlying surgical reports and subclassified in <200 ml blood loss and ≥200 ml blood loss. Moreover, the time of warm ischemia as well as the total surgery time were assessed.

Peri- and postoperative complications were classified according to the Dindo-Clavien-classification [14]. The cohort was subdivided in either one subgroup with (Clavien-Dindo grade >1) or without severe peri- or postoperative complications (Clavien-Dindo grade 0 or 1).

### Image evaluation

Only CT images that were acquired <3 months before the date of surgery were selected for image analysis. Due to different places of origin, the CT technique was slightly heterogeneous. However, all images were gained with modern multi-detector CT (MDCT), using a tube current of 100–120 kVp. Most scans were performed using a vendor-dependent, automated tube current modulation technique. The slice thickness for all CT images ranged between 1.5 and 5 mm. Images were processed with a vendor-dependent soft tissue kernel.

Since Lubner et al. have detected a maximum specificity for portovenous or venous CT images, only these were included in this study [8].

All CT images were transferred to dedicated tumor evaluation software with an integrated CT texture analysis application (mint Lesion^™^, v. 3.2, mint medical, Dossenheim, Germany). Afterwards, all images were evaluated by two radiologists, with 6 years of expertise in oncological imaging (J-M.H. and G.B.), who were informed about the localization of the tumor, but not about the histological report results. Using the implemented volume of interest (VOI) tool, both readers performed slice-by-slice segmentation of the tumors with automated contour interpolation. As reference, another VOI was drawn inside the renal cortex in an image slice centering the tumor. The tumor volume, long and short axis, the mean attenuation value and attenuation standard deviation were calculated by the software. Moreover, histogram-based texture analysis measures for skewness, kurtosis, Shannon’s entropy, uniformity, mean of positive pixels (MPP), and uniformity of distribution of positive gray-level pixel values (UPP) were assessed from both VOI using the software-specific settings.

To avoid examination-related artifacts, ratios between the tumor measures and the kidney cortex measures for mean attenuation, attenuation standard deviation, kurtosis, entropy, uniformity, MPP, and UPP, were calculated as follows:

$$Parameter\;ratio=\frac{{CT\;parameter\;value}_{tumor}}{{CT\;parameter\;value}_{kidney}}$$ (1)

Moreover, the skewness difference between tumor and kidney parenchyma (skewness_diff_) was calculated. To evaluate, whether these corrections are necessary, a correlation analysis between the raw values and the reference-corrected values was performed.

Afterwards, the predictive value of these texture analysis-based parameters for the tumor type and grade, as well as for the prediction of perioperative complications, was calculated.

### Statistical analysis

Statistical analysis was performed using the JMP 13.1 software package (SAS Institute Inc., Cary, NC, USA). Testing for normal distribution was performed using the Shapiro-Wilk-test.

CT texture parameters of clear cell RCC and non-clear cell RCC were compared to each other via analysis of variance (ANOVA) with a significance level of p < .05. For correlation analysis between raw measurement data and reference-corrected data, Spearman’s correlation coefficient ρ was calculated for all values. The strength of correlation was rated weak for ρ < .3, moderate for ρ≤.5 or strong for ρ>.5. Multivariate correlation analyses were performed for comparison of texture analysis parameters and warm ischemic time and surgery time with a corrected significance level of p = .01.

For diagnostic accuracy measures of these CT parameters, with focus on tumor grading and prediction of perioperative complications, logistic regression and receiver operating characteristic (ROC) curve analysis were performed for each CT parameter, and the area under curve (AUC) calculated. Youden’s index was used for the estimation of optimal cutoff values.

The intra-class correlation coefficient (ICC), as a measure for inter-reader agreement, was calculated using the Mangold Reliability calculator v. 1.5 (Mangold International GmbH, Arnstorf, Germany). The quality of agreement was defined as follows: slight agreement (ICC = 0–0.2), fair agreement (ICC = 0.21–0.4), moderate agreement (ICC = 0.41–0.6), substantial agreement (ICC = 0.61–0.8) and almost perfect agreement (ICC = 0.81–1.0).

## Results

### Patient cohort

Of the finally included 106 patients, 67 (63.2%) were male and 39 female (36.8%) with a median age of 65 years (range, 28–86 years). Detailed patient information is given in Table 1. Forty-seven of them presented with a histologically confirmed clear-cell RCC (69.8%) and 32 with a non-clear-cell RCC (30.2%). Altogether, 16 patients (15.1%) suffered severe perioperative complications. In 6 cases (5.7%) these were rated with the Clavien-Dindo grade II and in 5 cases each (4.7%) with a grade of III or IV.

**Table 1:** Patient characteristics.

Patient characteristic	n = 106	%
**Gender**		
Male	67	63.2
Female	39	36.8
**Median age (95%-CI)**	65 (61.7–66.3) years	
**Tumor type and grade**		
Clear cell RCC	74	69.8
Grade 1	33	31.1
Grade 2	38	35.8
Grade 3	3	2.9
Grade 4	0	0
Non-clear cell RCC	32	30.2
Grade 1	7	6.6
Grade 2	24	22.5
Grade 3	1	0.9
Grade 4	0	0
**Clavien-Dindo score**		
0	81	76.4
1	9	8.5
2	6	5.7
3	5	4.7
4	5	4.7
5	0	0

From the group with minor complications (Clavien-Dindo type I), all 9 needed pharmacological treatment (excluding blood transfusion). All patients with a Clavien-Dindo grade > 1 were sub-summarized as “severe complications”. All 6 patients with a Clavien-Dindo grade II needed a perioperative blood transfusion. Of the grade III complication group, 3/5 (60%) were referred to a radiological angiographic intervention because of a pseudoaneurysm, whilst 2/5 (40%) had a urinoma. Of the 5 patients with grade IV complications, 2/5 (40%) needed further intervention due to bowel injury and 3/5 (60%) were referred on an intensive care unit >20h after surgery.

### Image analysis results—Tumor type and grade

All results of the CT image and texture analysis are given in S1 Table. Statistically significant differences between clear-cell subtype of renal cell carcinomas (CC-RCC) and non-clear-cell subtype (NCC-RCC) imaging characteristics are summarized in Table 2 (all comparisons are listed in S2 Table). The references for the kidney parenchyma are given in the supporting information (S5 Table). The correlation analysis between the initial measurements and the reference-corrected data (ratios or difference) yielded the following results: tumor attenuation (ρ = .731; p = < .0001), tumor attenuation SD (ρ = .679; p = < .0001), skewness (ρ = -.896; p < .0001), kurtosis (ρ = .912; p < .0001), entropy (ρ = .554; p < .0001), uniformity (ρ = .582; p < .0001), MPP (ρ = .697; p < .0001), and UPP (ρ = .633; p < .0001).

**Table 2:** Significant different imaging characteristics between patients with clear-cell and non-clear-cell renal cell carcinoma.

Characteristic	Clear-cell subtype	Non-clear-cell subtype	p (uncorrected/corrected)
**Mean attenuation [HU]**			.017/ < .001
Mean±SD	89.36±37.47	71.94±23.49	
Median	86.55	66.03	
Range	12.65–190.2	23.95–128.7	
**Skewness**			.194/.028
Mean±SD	-0.25±0.42	-0.13±0.47	
Median	-0.28	-0.15	
Range	-1,15–1	-1.1–0.8	
**Entropy**			.031/.12
Mean±SD	6.7±0.37	6.54±0.26	
Median	6.61	6.5	
Range	5.75–7.59	6.08–7.13	
**MPP**			.015/ < .001
Mean±SD	90.68±36.3	73.51±22.42	
Median	86.98	67.7	
Range	22.05–190.2	37.45–128.7	
**UPP**			.023/.922
Mean±SD	0.012±0.003	0.015±0.009	
Median	0.012	0.013	
Range	0.006–0.027	0.008–0.061	

Abbreviations: MPP—mean of positive pixels; UPP—uniformity of distribution of positive gray-level pixel values.

The MPP value differed significantly between CC-RCC and NCC-RCC with a p-value of p = .015 for non-corrected and p < .0001 for reference-corrected values as well as the mean attenuation (p = .017/ < .0001). Regarding the other CT and texture analysis parameters, the skewness_diff_ was also significantly different (p = .028), but not the uncorrected skewness values (p = .194). In contrast, the difference between both tumor subtypes was significant when using the uncorrected entropy (p = .031 vs. p = .12 with reference-corrected values) or the uncorrected UPP values (p = .023 vs. p = .922 for reference-corrected values). Therefore, all further tests were performed with corrected and uncorrected values.

**Fig 2:**
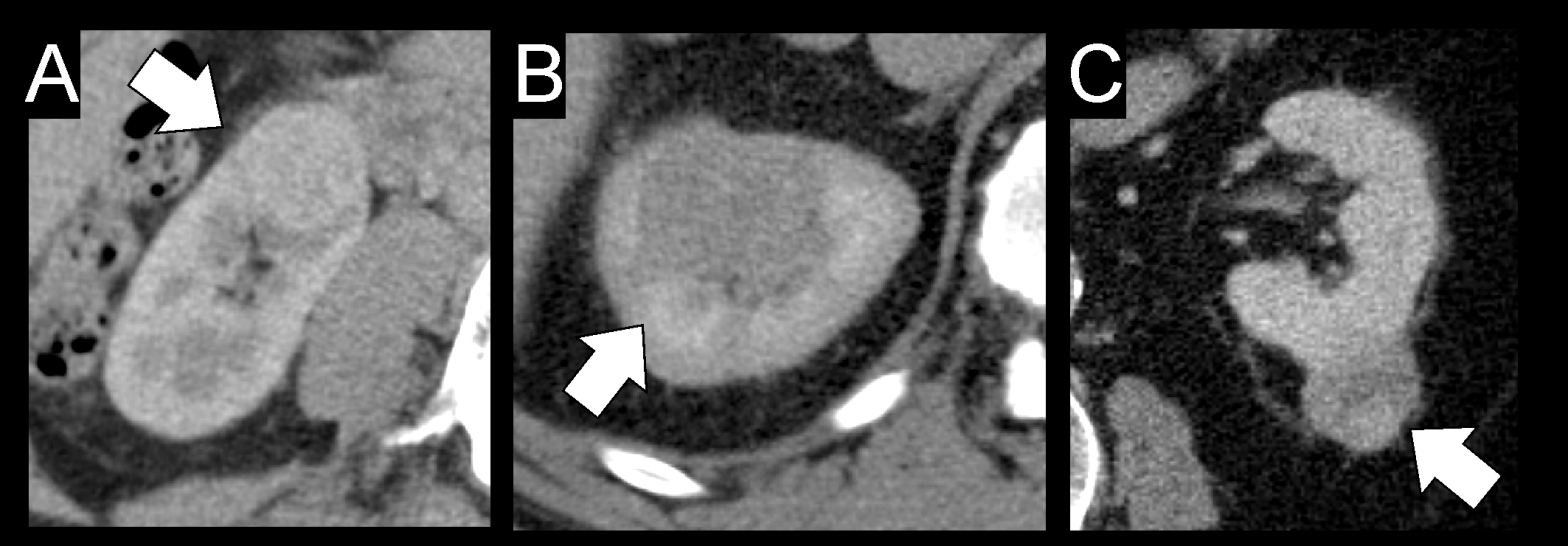
CT image examples of three different patients with renal cell carcinoma and different tumor nuclear grade. Whilst Fig 1A shows a grade 1 tumor of the right kidney of a 49 year-old female patient, Fig 1B is displaying a grade 2 clear-cell RCC of a 58 year-old male patient, and Fig 1C is from a 78 year-old male patient with a grade 3 tumor. All tumors are approximately the same size; however, a grading based on visual criteria only is not possible.

The results of the ROC analysis with the endpoints high-grade tumor (nuclear grade G2 and G3) and low-grade tumor (nuclear grade G1) are summarized in S3 Table (reference-corrected) and S4 Table (uncorrected data). Concurring from these results, only the uncorrected skewness values have a poor diagnostic accuracy regarding discrimination of higher and low grade RCC. An image example with three different tumor grades is given in Fig 2.

### Image analysis results—Perioperative outcome

**Fig 3:**
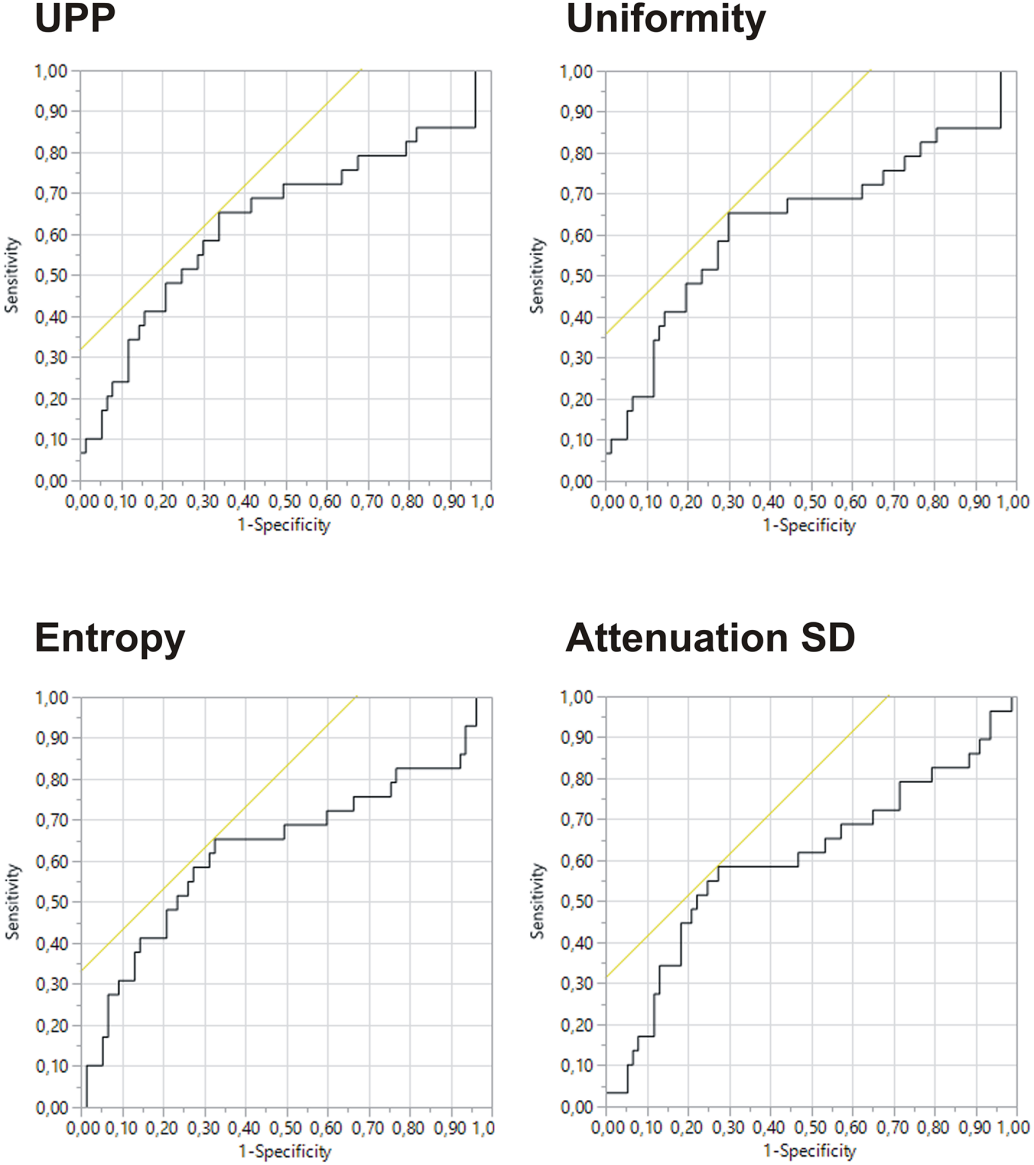
Receiver operating characteristic (ROC) curves for CT texture analysis parameters predicting a perioperative blood loss ≥200 ml. The figure includes all reference-corrected parameters with an area under curve >0.6 for prediction of an increased blood loss during surgery. Abbreviations: UPP, uniformity of positive pixel values; SD, standard deviation.

Of all included patients, 29 patients (27.4%) showed a blood loss of ≥200ml during surgery. The CT texture analysis with reference-corrected values yielded best results for the UPP (AUC, 0.638), followed by the uniformity (AUC, 0.635), the tumor entropy (AUC, 0.633) and the standard deviation of the HU attenuation (AUC, 0.605; see S6 Table and Fig 3). In most cases, the reference-corrected data was superior to the uncorrected regarding the prediction of increased blood loss. The tumor grade yielded an AUC of 0.574 and was also considered a weak predictor of perioperative blood loss.

**Fig 4:**
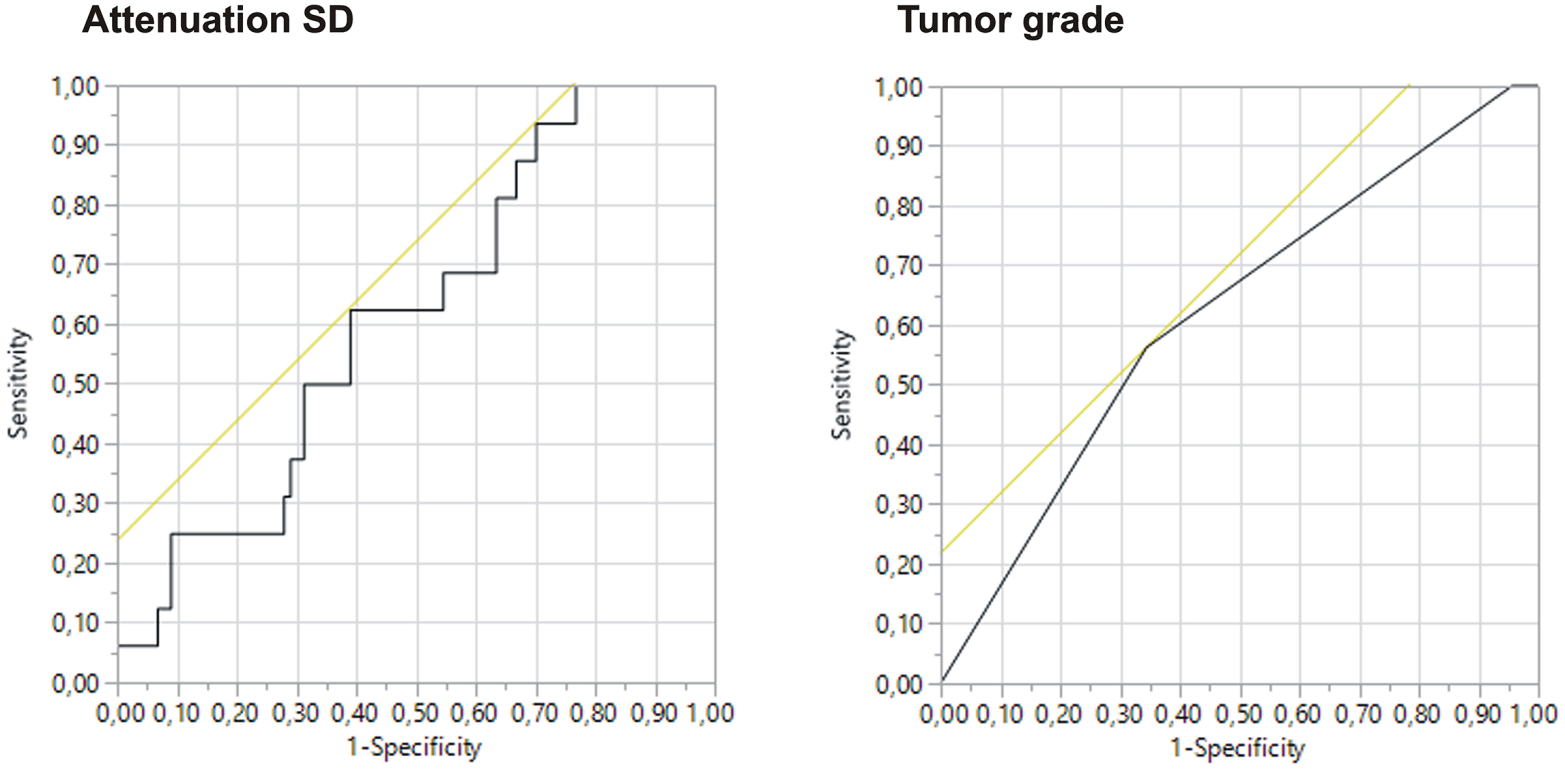
Receiver operating characteristic (ROC) curves for the standard deviation of CT attenuation values (attenuation SD) and tumor grade for prediction of perioperative complications. Under all reference-corrected values, the standard deviation of the CT attenuation was the only parameter with an AUC > 0.6. In comparison, the ROC curve for the tumor grade is given.

The results for the outcome analysis (unfavorable outcome: severe complications) including all tumor types are summarized in S7 Table. Of all reference-corrected texture analysis parameters, only the tumor/kidney-ratio of the attenuation SD presented with an AUC value > 0.6, compared to the uncorrected image data, where the mean attenuation (AUC, 0.615) and the MPP (AUC, 0.608) presented with a poor, but usable predictive value. The tumor diameter yielded an AUC of 0.469 (threshold, 34.15mm; sensitivity, 75.0%, specificity, 61.1%), whilst a tumor nuclear grade ≥G2 turned out to be the best predictive parameter for perioperative complications with an AUC of 0.619 (sensitivity, 56%, specificity, 65.6%; Fig 4).

The average time of surgery was 130.29±48.84 minutes and the average ischemic time during surgery was 14.97±11.44 minutes. The best, although weak and non-significant correlations were found between the time of surgery and the reference-corrected MPP (r = .2, p = .03) and the mean tumor attenuation (r = .23; p = .011). All other correlations were worse.

### Inter-reader agreement

The overall ICC with strict calculation of scores was .982 and therefore considered as almost perfect agreement.

## Discussion

In the presented study, we aimed to assess whether CT texture analysis of the internal structure of renal cell carcinomas is a valuable tool for prediction of perioperative complications during laparoscopic partial nephrectomy. On the one hand, we hypothesized that morphological inhomogeneity in RCC may correlate with a more complex inner tumor structure and may therefore be an additional risk factor for minimally invasive surgical approaches. To avoid technically-dependent bias and generate generally applicable results, all CT (including CT texture analysis) parameters were set in relation to the renal parenchyma.

Histogram-based texture analysis methods are being applied more frequently in imaging and pathological sciences for quantitative description of the structural homogeneity or heterogeneity of different body tissues end especially tumorous tissue, where structural inhomogeneity is a frequent “key feature of malignancy” [15]. For description of the homogeneity of grey-level distribution in a histogram (a display of the range and frequency of the pixel intensity values in an image or image section), different mathematical measures are applicable. For example, so called first-order statistics as the mean of the pixel values and the uniformity describe the probability distribution of individual pixel values. Another first-order statistic value is the entropy, which aims to describe the complexity of a given information quantitatively: simplified, a high entropy value means that the image section has a greater variance of grey level pixels [16, 17]. In our study, Shannon’s entropy was applied, which is based on the logarithm of the probability distribution of the grey-level pixels in the image section and is applied in most radiological image processing tools [18]. Higher-order statistics as the skewness as a measure for asymmetry of the histogram or the kurtosis as descriptor for the histogram curves’ shape have recently also been more frequently and successfully applied in tumor diagnosis [15, 19].

Regarding all applied parameters, the tumor/parenchyma-ratio of the HU standard deviation proved to be the most sensitive for prediction of perioperative complications (including perioperative blood loss), followed by the tumor uniformity, UPP and entropy. Moreover, all acquired parameters yielded a predictive value superior to the tumor diameter with an AUC < 0.5, which is knowingly one of the most predictive tumor features and therefore included in the R.E.N.A.L. nephrometry system or the centrality index (C-index) [5, 20, 21]. However, the tumor size alone is known to be a weaker predictor than scores using multiple morphological or morphometric parameters [22]. So, both scores add other parameters as for example localization inside the kidney tissue or nearness to vessels or the kidney pelvis. However, both scores are primarily based on the tumor localization and information about the inner tumor structure is not included. Other scores used for prediction of surgical outcome during or after LPN completely neglect the tumor itself, as exemplarily the MAP score, the PADUA score or the renal pelvic score [22–25].

A correlation between all calculated texture analysis values and the surgery time as well as the warm ischemic time was not found. However, these factors rely even more on the surgery team than only on the complexity of the tumor, which could explain the lack of correlation.

Recently, another scoring system, the arterial-based complexity (ABC) score has been established and successfully evaluated. This score focuses on the distance of the renal arteries and the tumor but also neglects the tumorous vessel structures [26]. This score as well does not include information about the inner tumor structure, which also reflects tumor vascularization and—considering the given results—represents an additional risk factor during surgery. Regarding this, histopathological analyses have already revealed, that RCCs show a different microvasculature, which correlates with the outcome, but is not part of the tumor grading, yet- This “increased microvascular density” is not characterized by arteriovenous shunting but represents a potential bleeding focus [13]. Therefore, the hypothesis is close, that texture analysis (especially in contrast enhanced images) may reflect the presence of these more vulnerable, immature vessel structures. Interestingly, the best parameters for prediction of blood loss during surgery were CT texture analysis parameters describing a relatively homogenous tumor attenuation (uniformity, UPP, entropy). However, further histopathological analysis focusing on the microvascular density, are needed for confirmation of this hypothesis.

Moreover, we evaluated whether the tumor nuclear grade, as a known predictor of poor long-term outcome, could be predicted by CT image and texture parameter analysis, which has been reported in RCC larger than 7 cm [8]. However, in our cohort, the CT texture analysis parameters with AUC values <0.6 did only poorly predict the nuclear grade in our cohort. One explanation for this difference between both studies is probably, that we included a comparably smaller tumor size in our cohort with a diameter range from 1.2–8.3 cm (mean, 3.3±1.5cm). Consecutively, this means, that CT texture analysis is not a valid tool for tumor grading in small RCCs, but some parameters are still valuable for outcome prediction, independently from the tumor grade. That makes CT texture a valuable parameter for preoperative risk stratification in small and large RCCs, but not for tumor grading in small RCCs [27, 28].

The reported inter-reader agreement in our study was almost perfect and higher than reported for other scoring systems [29–31], which underpins the reliability of the method. Nevertheless, it should be mentioned, that all presented CT imaging parameters do not suffice for a stand-alone predictive system and inclusion in a more complex score could reduce the overall reliability.

There are limitations of our study to be mentioned. First, the heterogeneity of the CT image assessment protocols is one of the major limitations. Especially skewness and kurtosis are parameters that are susceptible for variability among CT scanners [32]. To reduce this technically-derived bias and enhance generalizability, we included reference measurements of the kidney tissue that should homogenize the gained data. Since the correlation between non-corrected and reference-corrected values is good, but not perfect, we propose this reference-based approach for further generalizability and comparability of texture analysis studies. Second, the retrospective study design is an important limitation. Moreover, in contrast to other studies, only CT images gained in portovenous or venous contrast phase were evaluated, according to the results of Lubner et al. [8]. Last but not least, only a few surgical reports contained detailed information on the in-situ findings, which elsewise could have been correlated with the CT findings. To overcome these limitations, further prospective studies are recommended to validate our results.

## Conclusions

The presented CT and CT texture analysis parameters are useful parameters reflecting inner tumor structure alterations, which could enhance the value of existing scoring systems for prediction of perioperative outcome and intraoperative blood loss, during and after laparoscopic partial nephrectomy in renal cell carcinoma. A prediction of the tumor nuclear grade as a major predictive factor is only limitedly possible by these parameters in small tumors.

## Supporting information

S1 Table Summarized patient information. (XLSX)

S2 Table Imaging characteristics of patients with clear-cell and non-clear-cell renal cell carcinoma. (DOCX)

S3 Table Summary of the ROC curve analysis regarding high (nuclear grade G2 & G3) versus low nuclear grade (G1) with reference-corrected CT texture analysis data. (DOCX)

S4 Table Summary of the ROC curve analysis regarding high (nuclear grade G2 & G3) versus low nuclear grade (G1) with uncorrected CT texture analysis data. (DOCX)

S5 Table Reference values for the kidney parenchyma. (DOCX)

S6 Table Summary of the ROC curve analysis regarding the prediction of perioperative blood loss > 200ml with non-corrected and reference-corrected CT texture analysis data. (DOCX)

S7 Table Summary of the ROC curve analysis regarding the prediction of perioperative complications with non-corrected and reference-corrected CT texture analysis data. (DOCX)
