# Supplementary material for: Value of computed tomography texture analysis for prediction of perioperative complications during laparoscopic partial nephrectomy in patients with renal cell carcinoma
Source: PLoS One. 2018 Apr 18;13(4):e0195270. doi: 10.1371/journal.pone.0195270 (PMC5905959; doi:10.1371/journal.pone.0195270)
Supplement: S2 Table — (DOCX) [file pone.0195270.s002.docx]

| **Characteristic** | **Clear-cell subtype** | **Non-clear-cell subtype** | **p (uncorrected/corrected)** |
| --- | --- | --- | --- |
| Tumor volume [mm^3^]  Mean±SD  Median  Range | 20.39±30.41  8.53  0.75-184.6 | 19.04±20.82  9.68  0.55-71.25 | 0.82/0.84 |
| Long axis [mm]  Mean±SD  Median  Range | 33.42±14.85  28.55  12.45-83.05 | 31.37±13.61  29.1  10.65-54.85 | 0.506/0.64 |
| Short axis [mm]  Mean±SD  Median  Range | 26.53±12.02  22.75  9.35-68.8 | 26.17±12.69  24.73  8-50.15 | 0.887/0.98 |
| Mean attenuation [HU]  Mean±SD  Median  Range | 89.36±37.47  86.55  12.65-190.2 | 71.94±23.49  66.03  23.95-128.7 | 0.017/<0.001 |
| Attenuation SD [HU]  Mean±SD  Median  Range | 29.13±8.57  26.25  13.2-55.55 | 26.57±6.96  26.3  17.3-53.1 | 0.139/0.513 |
| Skewness  Mean±SD  Median  Range | -0.25±0.42  -0.28  -1,15-1 | -0.13±0.47  -0.15  -1.1-0.8 | 0.194/0.028 |
| Kurtosis  Mean±SD  Median  Range | 3.42±0.68  3.5  1.95-4.7 | 3.72±1.04  3.4  2.05-6.6 | 0.081/0.951 |
| Entropy  Mean±SD  Median  Range | 6.7±0.37  6.61  5.75-7.59 | 6.54±0.26  6.5  6.08-7.13 | 0.031/0.12 |
| Uniformity  Mean±SD  Median  Range | 0.012±0.003  0.012  0.006-0.027 | 0.013±0.003  0.013  0.008-0.018 | 0.087/0.858 |
| MPP  Mean±SD  Median  Range | 90.68±36.3  86.98  22.05-190.2 | 73.51±22.42  67.7  37.45-128.7 | 0.015/<0.001 |
| UPP  Mean±SD  Median  Range | 0.012±0.003  0.012  0.006-0.027 | 0.015±0.009  0.013  0.008-0.061 | 0.023/0.922 |

**S2 Table. Imaging characteristics of patients with clear-cell and non-clear-cell renal cell carcinoma.**

Abbreviations: MPP, mean of positive pixels; UPP, uniformity of distribution of positive gray-level pixel values; SD, standard deviation.
