# Supplementary material for: Value of computed tomography texture analysis for prediction of perioperative complications during laparoscopic partial nephrectomy in patients with renal cell carcinoma
Source: PLoS One. 2018 Apr 18;13(4):e0195270. doi: 10.1371/journal.pone.0195270 (PMC5905959; doi:10.1371/journal.pone.0195270)
Supplement: S3 Table — (DOCX) [file pone.0195270.s003.docx]

| **Characteristic** | **AUC** | **Threshold** | **Sensitivity [%]** | **Specificity [%]** |
| --- | --- | --- | --- | --- |
| CC-RCC  Mean attenuation*  Attenuation SD*  Skewness_diff_  Kurtosis*  Entropy*  Uniformity*  MPP*  UPP* | 0.548  0.564  0.561  0.541  0.54  0.56  0.554  0.567 | 0.69 HU  1.82 HU  0  0.95  1.2  0.47  0.67  0.47 | 82.9  63.4  39.0  31.7  61.0  61.0  75.6  61.0 | 33.3  57.3  78.8  81.82  37.6  60.6  42.4  60.6 |
| NCC-RCC  Mean attenuation*  Attenuation SD*  Skewness_diff_  Kurtosis*  Entropy*  Uniformity*  MPP*  UPP* | 0.474  0.554  0.578  0.549  0.52  0.497  0.526  0.509 | 0.59  1.49  0.1  1.21  1.17  0.515  0.59  0.583 | 16.0  44.0  48.0  60.0  36.0  44.0  24.0  32.0 | 100.0  83.7  71.4  71.43  83.7  71.4  100.0  85.7 |

**S3 Table. Summary of the ROC curve analysis regarding high (nuclear grade G2 & G3) versus low nuclear grade (G1) with reference-corrected CT texture analysis data.**

Abbreviations: AUC, Area under curve; CC-RCC: Clear cell renal cell carcinoma; MPP, mean of positive pixels; NCC-RCC, non-clear cell renal cell carcinoma; ROC, Receiver operating characteristic; SD, standard deviation; UPP, uniformity of distribution of positive gray-level pixel values.
