# Supplementary material for: Value of computed tomography texture analysis for prediction of perioperative complications during laparoscopic partial nephrectomy in patients with renal cell carcinoma
Source: PLoS One. 2018 Apr 18;13(4):e0195270. doi: 10.1371/journal.pone.0195270 (PMC5905959; doi:10.1371/journal.pone.0195270)
Supplement: S4 Table — (DOCX) [file pone.0195270.s004.docx]

| **Characteristic** | **AUC** | **Threshold** | **Sensitivity [%]** | **Specificity [%]** |
| --- | --- | --- | --- | --- |
| CC-RCC  Mean attenuation  Attenuation SD  Skewness  Kurtosis  Entropy  Uniformity  MPP  UPP | 0.559  0.521  0.575  0.513  0.562  0.555  0.561  0.575 | 92.6 HU  24.9 HU  -0.5  2.85  6.6  0.009  92.6  0.012 | 65.9  41.5  34.2  82.9  56.1  85.4  65.9  61.0 | 57.5  65.7  81.8  24.2  60.6  30.3  57.6  54.5 |
| NCC-RCC  Mean attenuation  Attenuation SD  Skewness  Kurtosis  Entropy  Uniformity  MPP  UPP | 0.537  0.566  0.617  0.566  0.589  0.586  0.491  0.437 | 66.25  28.2  0.0  3.15  6.6  0.012  101.8  0.016 | 60.0  80.0  72.0  80.0  48.0  44.0  20.0  28.0 | 71.4  57.1  71.4  42.8  85.7  85.7  100.0  85.7 |

**S4 Table. Summary of the ROC curve analysis regarding high (nuclear grade G2 & G3) versus low nuclear grade (G1) with uncorrected CT texture analysis data.**

Abbreviations: AUC, Area under curve; CC-RCC: Clear cell renal cell carcinoma; MPP, mean of positive pixels; NCC-RCC, non-clear cell renal cell carcinoma; ROC, Receiver operating characteristic; SD, standard deviation; UPP, uniformity of distribution of positive gray-level pixel values.
