# Supplementary material for: Value of computed tomography texture analysis for prediction of perioperative complications during laparoscopic partial nephrectomy in patients with renal cell carcinoma
Source: PLoS One. 2018 Apr 18;13(4):e0195270. doi: 10.1371/journal.pone.0195270 (PMC5905959; doi:10.1371/journal.pone.0195270)
Supplement: S5 Table — (DOCX) [file pone.0195270.s005.docx]

| **Characteristic** | **Kidney reference value** |
| --- | --- |
| Mean attenuation [HU]  Mean±SD  Median  Range | 151.84±40.21  149.1  75.7-280.95 |
| Attenuation SD [HU]  Mean±SD  Median  Range | 15.83±3.7  15.15  8-26.85 |
| Skewness  Mean±SD  Median  Range | -0.04±0.19  0.0  -0.6-0.6 |
| Kurtosis  Mean±SD  Median  Range | 2.95±0.33  2.88  2.4-4.25 |
| Entropy  Mean±SD  Median  Range | 5.75±2.2  5.56  4.09-27.94 |
| Uniformity  Mean±SD  Median  Range | 0.026±0.009  0.025  0.016-0.099 |
| MPP  Mean±SD  Median  Range | 151.69±40.35  150.38  75.7-280.95 |
| UPP  Mean±SD  Median  Range | 0.026±0.009  0.025  0.016-0.099 |

**S5 table. Reference values for the kidney parenchyma.**

Abbreviations: MPP, mean of positive pixels; UPP, uniformity of distribution of positive gray-level pixel values; SD, standard deviation.
