# Supplementary material for: Value of computed tomography texture analysis for prediction of perioperative complications during laparoscopic partial nephrectomy in patients with renal cell carcinoma
Source: PLoS One. 2018 Apr 18;13(4):e0195270. doi: 10.1371/journal.pone.0195270 (PMC5905959; doi:10.1371/journal.pone.0195270)
Supplement: S6 Table — (DOCX) [file pone.0195270.s006.docx]

| **Characteristic** | **AUC** | **Threshold** | **Sensitivity [%]** | **Specificity [%]** |
| --- | --- | --- | --- | --- |
| Uncorrected data  Mean attenuation  Attenuation SD  Skewness  Kurtosis  Entropy  Uniformity  MPP  UPP | 0.533 0.5 0.514 0.566 0.483 0.54 0.528 0.556 | 99.5 30.8 -0.15 3.5 6.84 0.009 92.6 0.009 | 41.4 44.8 48.3 65.5 34.4 27.6 48.3  27.6 | 72.6 71.4 61.0 53.2 77.0 87.0 64.9 85.7 |
| Corrected data  Mean attenuation  Attenuation SD  Skewness_diff_  Kurtosis  Entropy  Uniformity  MPP  UPP | 0.489 0.605 0.528 0.564 0.633 0.635 0.474 0.638 | 0.51  1.99 0.1 1.21 1.21 0.432 0.42 0.432 | 75.9 58.6 55.2 69.0 65.5 65.5 89.7 65.5 | 35.1 72.7 58.4 50.6 67.5 70.1 19.4 66.2 |

**S6 Table.** Summary of the ROC curve analysis regarding the prediction of perioperative blood loss > 200ml with non-corrected and reference-corrected CT texture analysis data.
*Data calculated as ratio between tumor VOI measurement and kidney parenchyma VOI measurement.
Abbreviations: AUC, Area under curve; SD, standard deviation; UPP, uniformity of distribution of positive gray-level pixel values; VOI, volume of interest.
