# Supplementary material for: Value of computed tomography texture analysis for prediction of perioperative complications during laparoscopic partial nephrectomy in patients with renal cell carcinoma
Source: PLoS One. 2018 Apr 18;13(4):e0195270. doi: 10.1371/journal.pone.0195270 (PMC5905959; doi:10.1371/journal.pone.0195270)
Supplement: S7 Table — (DOCX) [file pone.0195270.s007.docx]

| **Characteristic** | **AUC** | **Threshold** | **Sensitivity [%]** | **Specificity [%]** |
| --- | --- | --- | --- | --- |
| Uncorrected data  Mean attenuation  Attenuation SD  Skewness  Kurtosis  Entropy  Uniformity  MPP  UPP | 0.615  0.519  0.545  0.508  0.561  0.566  0.608  0.572 | 101.0  28.75  -0.65  4.45  7.05  0.009  101.15  0.009 | 68.8  50.0  100.0  25.0  31.25  37.5  68.8  37.5 | 66.7  65.6  15.6  90.0  88.9  87.8  75.6  86.7 |
| Corrected data  Mean attenuation*  Attenuation SD*  Skewness_diff_  Kurtosis*  Entropy*  Uniformity*  MPP*  UPP* | 0.558  0.615  0.529  0.52  0.588  0.599  0.55  0.597 | 0.674  1.39  0.35  1.68  1.21  0.44  0.774  0.432 | 43.8  93.8  81.3  18.8  62.5  62.5  25.0  62.5 | 77.7  30.0  37.8  94.4  60.0  60.0  95.6  61.1 |

**S7 Table.** Summary of the ROC curve analysis regarding the prediction of perioperative complications with non-corrected and reference-corrected CT texture analysis data.
*Data calculated as ratio between tumor VOI measurement and kidney parenchyma VOI measurement.
Abbreviations: AUC, Area under curve; SD, standard deviation; UPP, uniformity of distribution of positive gray-level pixel values; VOI, volume of interest.
